# Hashimoto's Encephalopathy With Progressive Cognitive Decline: A Rare Autoimmune Disorder in Central America

**DOI:** 10.7759/cureus.83235

**Published:** 2025-04-30

**Authors:** Anthony Hong, Ana B Santos, Luis Fernando Moya Porras, Ana Lucía Piedra Pacheco, Isaac Hong, Jose E Esquivel, José D Villegas

**Affiliations:** 1 Faculty of Medicine, University of Costa Rica, San José, CRI; 2 Department of Endocrinology, Hospital San Juan de Dios, San José, CRI; 3 Department of Neurology, Hospital San Juan de Dios, San José, CRI

**Keywords:** anti-thyroglobulin antibodies, anti-thyroid peroxidase antibodies, autoimmune, encephalopathy, hashimoto

## Abstract

Hashimoto's encephalopathy (HE) is a rare autoimmune disorder, typically associated with Hashimoto's thyroiditis, and often presents with rapidly progressive cognitive impairment, psychiatric symptoms, and neurological deficits. Since the first case reported, there have been relatively few reports of HE cases, and it remains rare in Central America. A 59-year-old male patient with a past medical history of hypertension (HTN) and type 2 diabetes mellitus (DM2) was hospitalized due to a one-month history of rapidly progressive cognitive impairment associated with behavioral changes and episodes of amnesia. It was not associated with motor or sensory deficits, seizures, or movement disorders. Given this presentation, alongside normal head CT findings and mild age-related brain atrophy on MRI, the initial diagnosis posed a challenge. Further investigations, including cerebrospinal fluid (CSF) analysis, autoimmune panels, and viral encephalitis testing, helped exclude other potential causes of encephalopathy. Notably, elevated anti-thyroid peroxidase (anti-TPO) and anti-thyroglobulin (anti-TG) antibodies were detected, strongly suggesting HE. The patient responded favorably to immunosuppressive treatment with intravenous methylprednisolone and subsequent oral prednisone, leading to significant cognitive improvement and recovery of function. This case highlights the importance of considering HE in the differential diagnosis of rapidly progressive cognitive decline, especially when routine standard investigations do not reveal an alternative cause.

## Introduction

Hashimoto's encephalopathy (HE), also known as steroid-responsive encephalopathy associated with autoimmune thyroiditis (SREAT), is a rare autoimmune disorder associated with Hashimoto's thyroiditis, characterized by a range of neurological and psychiatric symptoms [1]. Since its initial description by Brain et al. in 1966, HE has remained a rare diagnosis, with a relatively small number of cases reported worldwide [2,3]. It typically presents with rapidly progressive cognitive decline, psychiatric changes, seizures, and various neurological deficits, making its diagnosis challenging and often leading to delays in treatment [4]. Although the disease predominantly affects women, it can occur in both sexes, with an average onset age of around 38 years [5].

While the exact pathophysiology of HE is still not fully understood, it is believed to result from autoimmune-mediated brain inflammation. The diagnosis is based on a combination of clinical features, elevated thyroid antibodies, exclusion of other causes, and response to immunotherapy. Elevated thyroid antibodies, such as anti-thyroid peroxidase (anti-TPO) and anti-thyroglobulin (anti-TG), are commonly found in affected individuals and serve as important diagnostic markers. The autoimmune nature of HE is further supported by its association with other immunologic disorders, such as myasthenia gravis, glomerulonephritis, primary biliary cirrhosis, pernicious anemia, and rheumatoid arthritis. Inflammatory changes in the cerebrospinal fluid (CSF) and the condition's responsiveness to steroid treatment also lend evidence to its autoimmune etiology [1].

Notably, while cases have been reported across various continents, to the best of our knowledge, this is the first reported case in Central America to date. This case report illustrates the clinical course of a 59-year-old male patient with rapidly progressive cognitive decline. It not only adds to the limited global literature on HE but also emphasizes the critical need for heightened clinical suspicion in regions where the condition remains underreported, potentially leading to underdiagnosis and delayed treatment.

## Case presentation

A 59-year-old male patient with a medical history significant for hypertension (HTN), type 2 diabetes mellitus (DM2), and a left infracondylar amputation secondary to DM2. His current medications were aspirin 100 mg daily, irbesartan 300 mg daily for hypertension, neutral protamine Hagedorn (NPH) insulin 10-0-40 units for DM2, carbamazepine 200 mg three times a day for seizure control, gabapentin 600 mg three times a day for neuropathic pain, and sertraline 50 mg daily for anxiety and depression. The patient presented to the emergency room (ER) with a history of progressive cognitive decline over a period of one month. His cognitive difficulties initially manifested as forgetfulness of everyday tasks, such as losing track of where he was going. His memory problems progressively worsened, eventually leading to complete dependence on others for daily activities. In addition to cognitive impairment, he also developed urinary incontinence and frequent disorientation. The patient reported a history of frequent headaches since adolescence but denied any recent head trauma, chest pain, seizures, or fever.

In the ER, the patient's vital signs were recorded as normal: blood pressure of 130/80 mmHg, heart rate of 76 beats per minute, respiratory rate of 18 breaths per minute, and temperature of 36.7°C, without evidence of acute distress. Upon initial examination, the patient was alert and conscious. He was oriented to his name but was unable to comprehend the purpose of the consultation. His speech was fragmented and incoherent, indicative of aphasia. The patient's eye examination revealed symmetric pupils with normal pupillary light reflex. The cranial nerve examination revealed no deficits in the function of cranial nerves I-XII. There was no evidence of facial weakness, ptosis, or dysphagia, but signs of aphasia were noted, particularly in understanding speech. Muscle strength was normal in the upper and lower limbs (5/5), and no signs of atrophy or weakness were observed. Deep tendon reflexes were normal (2+ in all tested limbs), and there were no pathological reflexes observed. Sensation to light touch, pain, temperature, and proprioception was intact in all extremities. The oral cavity examination showed no oral lesions or apparent oral motor dysfunction. His neck was supple, with no signs of rigidity or masses. Skin examination revealed no changes, and the skin was warm, dry, and intact.

Given the patient's rapidly progressive cognitive decline and neurological findings, a comprehensive workup was initiated to explore potential causes. His Montreal Cognitive Assessment (MOCA) score was 17 points. Laboratory tests revealed several essential findings (Table 1). Thyroid function tests showed normal TSH levels, but T4 was in the lower limit of the normal range, and thyroid antibody tests were significantly elevated. Anti-TPO was 1,383 IU/mL (reference range: <35 IU/mL), and anti-TG was 48.5 IU/mL (reference range: <4 IU/mL), all of which were markedly higher than the normal reference range, suggesting an underlying autoimmune thyroid disorder. Coagulation studies were normal, and a complete blood count showed no significant abnormalities. Renal function and liver enzymes were all within normal limits, but with slightly elevated liver enzymes (aspartate aminotransferase (AST) and alanine aminotransferase (ALT)) and hyponatremia, but within non-critical ranges. The lipid profile showed elevated triglycerides and low-density lipoprotein (LDL) levels.

**Table 1:** Initial laboratory tests of the patient BUN: blood urea nitrogen, TSH: thyroid-stimulating hormone, HDL: high-density lipoproteins, LDL: low-density lipoproteins, AST: aspartate aminotransferase, ALT: alanine transaminase, ALP: alkaline phosphatase, GGT: gamma-glutamyl transferase, TPO: thyroid peroxidase, TG: thyroglobulin

Laboratories	Results	Reference range
Hemoglobin (g/dL)	14	13-17
Platelets	198,000	150,000-450,000
Leukocytes	5,500	4,000-10,000
BUN (mg/dL)	18	7-20
Creatinine (mg/dL)	0.95	0.7-1.3
Sodium (mEq/L)	130	136-145
Potassium (mEq/L)	3.9	3.5-5.0
Chloride (mEq/L)	107	98-107
TSH (µU/mL)	2.05	0.4-4
T4 (ng/dL)	0.89	0.8-1.8
Anti-TPO (IU/mL)	1,383	<35
Anti-TG (IU/mL)	48.5	<4
Vitamin B12 (pg/mL)	203	200-900
Triglycerides (mg/dL)	181	<150
Total cholesterol (mg/dL)	225	<200
HDL (mg/dL)	45	>40
LDL (mg/dL)	143	<100
ALT (UI/L)	50	7-56
AST (UI/L)	43	10-40
GGT (UI/L)	87	9-48
ALP (UI/L)	67	44-147

CSF analysis, which was performed twice, revealed normal white blood cells and mild elevation in protein levels (Table 2). Both CSF analyses showed no pleocytosis, and tests for infections, including the Venereal Disease Research Laboratory (VDRL) test, India ink, and cultures for bacteria, fungi, and mycobacteria, were all negative. There were no oligoclonal bands in the CSF, and autoimmune encephalitis panels were also negative, ruling out other possible causes of encephalitis. These findings, in combination with the significantly elevated thyroid antibodies, helped confirm the diagnosis and exclude other causes of encephalopathy, such as infections, malignancies, metabolic, or vascular disorders.

**Table 2:** CSF analysis results and normal reference ranges CSF: cerebrospinal fluid

Laboratories	At initial presentation	After 15 days of hospitalization	Reference range
Pressure (cmH_2_O)	32	20	5-20
Appearance	Clear	Clear	Clear
Protein (mg/dL)	50	49	12-45
Glucose (mg/dL)	69 (serum: 135)	141 (serum: 319)	40-70
White blood cell count (cells/mm^3^)	0	1	0-5
Red blood cells (cells/mm^3^)	1	0	None reported

**Figure 1:**
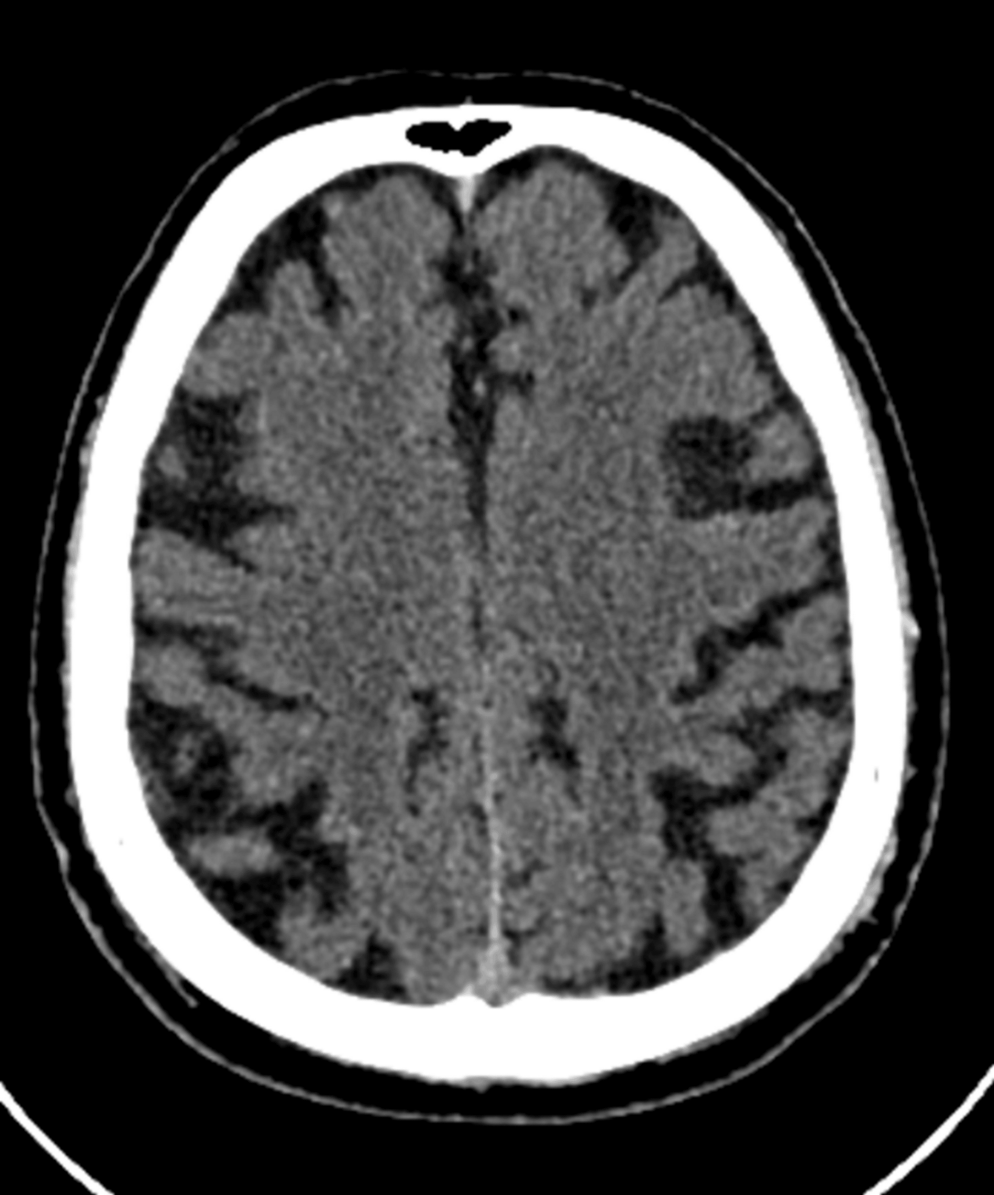
Axial CT scan image of the brain showing mild cortical atrophy

**Figure 2:**
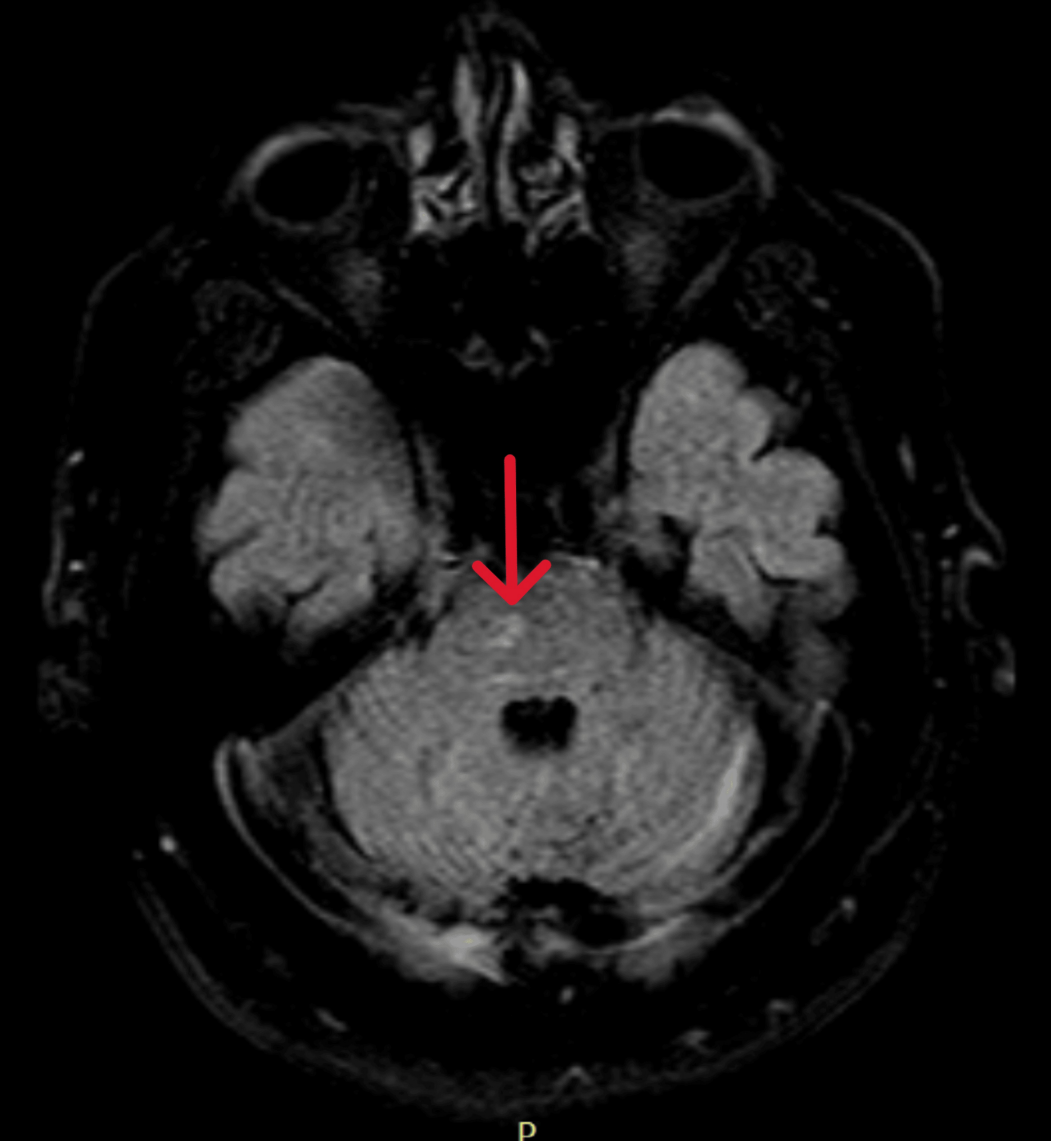
Axial FLAIR MRI of the brain showing a small right pontine lacunar infarct and cortical atrophy (arrow) FLAIR: fluid-attenuated inversion recovery

Imaging studies were performed to investigate possible structural causes for the patient's cognitive decline. A CT scan showed signs of involutional changes, but no evidence of acute pathology (Figure 1). MRI revealed small vessel disease, diffuse cortical atrophy, and a small pontine lacunar infarct. No acute vascular lesions were identified (Figure 2). An EEG showed diffuse slowing, but no epileptiform activity was noted.

After reviewing the clinical presentation, laboratory results, and imaging findings, the diagnosis of Hashimoto's encephalopathy was considered. The patient had elevated thyroid antibodies and a rapid onset of cognitive and neurological symptoms, which fit the clinical profile of this rare condition. The diagnosis was further supported by the exclusion of other causes for his symptoms, including autoimmune encephalitis, central nervous system malignancy, and other metabolic or infectious disorders.

Once the diagnosis was established, treatment was promptly initiated with a combination of intravenous immunoglobulin (IVIg) and high-dose corticosteroids. The patient received 40 g/day of IVIg for five days and 1 g/day of methylprednisolone for five days. Subsequently, a tapering regimen of oral prednisolone was started at 30 mg daily, with gradual reductions over the course of several months. In addition to these treatments, the patient continued his regular medications for managing his comorbidities.

The patient showed marked improvement after starting treatment. His cognitive function improved significantly, as demonstrated by an increase in his MOCA score from 17 points (indicating moderate cognitive impairment) to 21 points after therapy. His aphasia resolved, and he regained independence in daily activities. The patient has been followed up regularly and has not experienced any new symptoms.

## Discussion

This case presents a 59-year-old male patient with progressive cognitive decline, urinary incontinence, and neurological deficits, who was ultimately diagnosed with HE. The diagnosis was supported by elevated thyroid antibodies, particularly anti-TPO and anti-TG, along with the exclusion of other potential causes of encephalopathy, such as infections, malignancies, or metabolic disorders.

HE is a rare condition with an estimated prevalence of 2.1/100,000 in the adult population [6]. It is characterized by the onset of neurological symptoms in patients with Hashimoto's thyroiditis. These symptoms can include cognitive dysfunction, seizures, myoclonus, movement disorders, and psychiatric symptoms [4]. The patient's cognitive decline, which initially manifested as forgetfulness and disorientation and later progressed to severe dependency on others for daily tasks, is consistent with the typical course of HE. Cognitive dysfunction is one of the most prominent features of HE, and while it is often seen as progressive, it can vary in severity depending on the individual [1].

Interestingly, the patient in this case is male, which deviates from the typically female-dominant epidemiological profile of HE. Studies suggest that HE predominantly affects women, with a male-to-female ratio of about 1:4 [5], making this a remarkable aspect of the case. Furthermore, the patient's age (59 years) is slightly older than the typical onset age of HE, which usually occurs between 30 and 40 years [5]. This could suggest that HE can present later in life, although the disease remains rare in older populations.

The diagnosis of HE remains controversial and is typically considered a diagnosis of exclusion [5]. Graus et al.'s 2016 diagnostic framework outlines that HE requires clinical features such as encephalopathy with symptoms such as seizures, myoclonus, hallucinations, or stroke-like episodes, combined with elevated thyroid antibodies and a positive response to steroids [7]. This patient met several of these criteria, including progressive cognitive decline, nonspecific MRI abnormalities, markedly elevated thyroid antibodies, and response to steroids. The absence of other neuronal antibodies and the exclusion of alternative encephalopathies further support the diagnosis.

The diagnosis of HE is challenging due to the presence of elevated thyroid antibodies in 95%-100% of cases, even in the absence of significant thyroid dysfunction [8]. HE can occur in patients with normal thyroid function, and approximately 42% of individuals are euthyroid at the time of diagnosis [9]. In this case, despite the patient maintaining euthyroid status, anti-TPO levels were significantly elevated at 1,383 IU/mL (reference range: <35 IU/mL) and anti-TG levels at 48.5 IU/mL (reference range: <4 IU/mL), highlighting the importance of these elevated antibody levels as a key diagnostic indicator.

CSF analysis revealed a mild elevation in protein levels (50 mg/dL) with a normal white blood cell count and glucose, as well as negative tests for infections, autoimmune encephalitis, and malignancy. These CSF findings are consistent with those seen in HE, as elevated protein levels without significant pleocytosis are a common feature [1]. The absence of other inflammatory markers in the CSF, combined with negative infectious and autoimmune encephalitis panels, helps rule out other encephalopathies.

The MRI and CT scans of the brain were nonspecific, showing chronic changes such as small vessel disease, cortical atrophy, and a lacunar infarct. These findings, although commonly seen in the aging population, do not fully explain the patient's acute cognitive and neurological symptoms.

The differential diagnosis of HE is broad and complex, as its clinical manifestations can mimic a variety of neurological and psychiatric conditions, including delirium, rapidly progressive dementia, seizures, and focal neurological deficits. This overlap makes distinguishing HE from other disorders challenging. Conditions such as stroke, transient ischemic attack, cerebral vasculitis, carcinomatous meningitis, and toxic metabolic encephalopathies should be considered in the differential diagnosis [1]. Additionally, paraneoplastic syndromes, Creutzfeldt-Jakob disease, neurodegenerative diseases such as Alzheimer's and frontotemporal dementia, and psychiatric disorders including psychosis, mania, and severe depression can present with similar clinical features [6]. Other relevant conditions to consider include acute disseminated encephalomyelitis, cerebral vasculitis, and transient global amnesia, further complicating the diagnostic process [1]. In this case, the patient's symptoms closely resembled those of HE.

As this case illustrates, while structural neuroimaging can occasionally show nonspecific abnormalities in patients with HE, its diagnostic value is often limited. The diagnosis of HE primarily relies on clinical evaluation, which is based on the observation of neuropsychiatric symptoms, elevated levels of antithyroid antibodies, and the critical step of ruling out other potential causes of encephalopathy. This emphasizes the importance of maintaining a high level of suspicion and adopting a thorough diagnostic strategy [5].

Treatment of HE primarily involves corticosteroids, which are effective in most cases. However, only about 50% of patients respond to corticosteroid therapy [5]. In this case, the patient responded favorably to high-dose methylprednisolone and IVIg, demonstrating a significant improvement in cognitive function, as reflected in his MOCA score improving from 17 to 21 points, as well as regaining functionality and independence for daily activities. The prognosis of HE is highly variable, with patients typically experiencing either a relapsing-remitting or progressive course. While glucocorticoid therapy leads to symptom improvement in the majority of cases, a substantial number of patients may improve without steroid treatment [1]. Among patients who respond to treatment, approximately 12.5% experience relapses, while 12.5% exhibit no response at all. The remaining 60% experience a relapsing-remitting course [9]. Additionally, patients with higher initial anti-TPO antibody titers often experience more favorable outcomes, highlighting the potential prognostic value of these markers in predicting the disease's progression [9].

## Conclusions

This case of Hashimoto's encephalopathy in a 59-year-old male patient emphasizes the diagnostic complexities of this rare autoimmune disorder. The patient's progressive cognitive decline, along with significantly elevated thyroid antibodies in the absence of overt thyroid dysfunction, was pivotal in reaching the diagnosis. This case highlights the importance of considering HE in the differential diagnosis of rapidly progressing encephalopathy, even when thyroid function appears normal. Elevated anti-TPO and anti-TG antibodies serve as essential markers in identifying HE, and the patient's favorable response to corticosteroid and IVIg treatment reinforces the efficacy of early intervention. By presenting this case, we aim to raise awareness of HE, particularly in regions of Central America, and highlight the importance of timely diagnosis and treatment in regions where the condition may be overlooked, ultimately improving patient outcomes and advancing the understanding of this rare disorder.
